# The impact of SLCO1B1 rs4149056 on LDL-C target achievement after lipid lowering therapy optimization in men and women with familial hypercholesterolemia

**DOI:** 10.3389/fendo.2024.1346152

**Published:** 2024-02-08

**Authors:** Giosiana Bosco, Francesco Di Giacomo Barbagallo, Maurizio Di Marco, Nicoletta Miano, Sabrina Scilletta, Salvatore Spampinato, Alessio Vitale, Federica Di Bella, Maria Montalbano, Stefania Di Mauro, Agnese Filippello, Alessandra Scamporrino, Agostino Milluzzo, Antonino Di Pino, Lucia Frittitta, Francesco Purrello, Salvatore Piro, Roberto Scicali

**Affiliations:** Department of Clinical and Experimental Medicine, University of Catania, Catania, Italy

**Keywords:** familial hypercholesterolemia, LDL-C target, lipid lowering therapy, SLCO1B1 rs4149056, cardiovascular risk

## Abstract

**Background and aims:**

FH women are less likely to receive intensive statin treatment and to obtain a 50% reduction of LDL-C from baseline compared to men with FH. SLCO1B1 rs4149056 might influence statin therapy compliance and thus LDL-C target achievement. Our aim was to evaluate the impact of SLCO1B1 rs4149056 on LDL-C target achievement after lipid lowering therapy (LLT) optimization in men and women with FH.

**Methods:**

This was a retrospective observational study involving 412 FH subjects with a probable or defined clinical diagnosis of FH who had had genetic analysis from June 2016 to September 2022. Biochemical analysis was obtained from all subjects at baseline and at the last follow-up after LLT optimization.

**Results:**

After LLT optimization the percentage of FH subjects on high-intensity statins decreased from the M/SLCO1B1- group to the W/SLCO1B1+ group and the same was found in LDL-C target distribution (for both *p* for trend < 0.01). The prevalence of SASE fear increased from the M/SLCO1B1- group to the W/SLCO1B1+ group and the same was observed in reported myalgia distribution (for both *p* for trend < 0.01). Logistic regression analysis showed that the W/SCLO1B1-, M/SCLO1B1+ and W/SCLO1B1+ groups were inversely associated with LDL-C target achievement (*p* for trend < 0.001) and the W/SCLO1B1+ group exhibited the strongest association.

**Conclusion:**

A low prevalence of FH women with SLCO1B1 rs4149056 were on high intensity statins and they rarely achieved LDL-C target. The genotype effect of SLCO1B1 rs4149056 could be more pronounced in FH women than men.

## Introduction

Familial hypercholesterolemia (FH) is the most frequent monogenic lipid disorder characterized by an increased plasma level of low-density lipoprotein cholesterol (LDL-C) since childhood (1). FH subjects have a high risk of premature atherosclerotic cardiovascular disease (ASCVD) mainly due to a lifelong elevated LDL-C plasma level that promotes the development and the progression of atherosclerotic injury in the arterial wall (2); however, among FH subjects ASCVD risk is highly heterogeneous and it seems to be also influenced by other risk factors beyond LDL-C (3).

Early diagnosis and lipid lowering treatment optimization can considerably reduce the risks of premature atherosclerotic cardiovascular disease (ASCVD) in FH subjects (4). Statin treatment is the cornerstone of lipid-lowering therapies (LLT) and it should be initiated as soon as possible, even during childhood, as it has been shown to decrease the risk of CVD in adults (5, 6). Although the efficacy and safety of statins have already been demonstrated (7), statin treatment discontinuation is frequent in clinical practice, especially among patients on high intensity or long-term statin therapies (8, 9). The most frequently observed disorder is the onset of statin associated muscle symptoms (SAMS) that leads to statin discontinuation (10); moreover, a higher SAMS prevalence was reported in women than in men and this could be explained by the different effects of gender on pharmacokinetics and pharmacodynamics of statins (11). Previous findings from the CAscade SCreening for Awareness and DEtection of Familial Hypercholesterolemia (CASCADE-FH) registry reported that FH women were less likely to receive an intensive statin treatment as well as not obtaining a 50% reduction from baseline LDL-C compared to FH men (12). Thus, a different statin approach could partially explain the high percentage of premature ASCVD reported both in men and women with FH in contrast to the sex related cardiovascular injury onset observed in the general population (13). Beyond the impact of sex on LLT use, genetic polymorphisms associated with statin trafficking into the liver might influence the adherence as well as the efficacy of statins (14).

The solute carrier organic anion transporter 1B1 (*SLCO1B1*) gene encodes organic anion transporter polypeptide 1b1 (OATP1B1) that carries statins into tissues (15). It has been shown that the single nucleotide polymorphism SLCO1B1 521T>C (rs4149056) enhanced statin plasma levels and it was associated with an increased risk of SAMS in the general population (16). However, the liver concentration of statins as well as their LDL-C lowering effect are reduced in subjects with SLCO1B1 rs4149056 (17). Thus, the achievement of the recommended LDL-C target could be difficult in FH subjects with SLCO1B1 rs4149056. There is no data regarding the impact of SLCO1B1 rs4149056 on LDL-C target achievement in FH subjects.

In this study we aimed to evaluate the impact of SLCO1B1 rs4149056 on LDL-C target achievement after lipid lowering therapy optimization in men and women with FH.

## Methods

### Study design and population

This was a retrospective observational study involving subjects with a probable or defined clinical diagnosis of FH (Dutch Lipid Clinical Network score ≥ 6) who had had genetic analysis (18) from June 2016 to September 2022. All subjects were enrolled from the referral lipid center of the University Hospital of Catania and were aged between 18 and 70 years at the time of enrollment. At baseline, all participants underwent a physical examination and review of their clinical history. All subjects had biochemical analysis at baseline and at the last follow-up (January 2023-June 2023) after at least 3 month’s lipid lowering therapy optimization that was performed according to LDL-C values as well as a physician’s decision and Italian reimbursement rules. According to 2019 ESC/EAS guidelines for the management of dyslipidemias, all FH subjects obtained lipid lowering therapy optimization that was defined as a daily intake of high intensity statins plus ezetimibe +/- proprotein convertase subtilisin/kexin type 9 monoclonal antibodies (PCSK9-mAb). Based on the recommendations of the ESC/EAS guidelines for the management of dyslipidemias, baseline LDL-C target was defined as the following: LDL-C < 70 mg/dL or < 100 mg/dL in FH subjects with or without ASCVD enrolled from June 2016 to August 2019 or LDL-C < 55 mg/dL or < 70 mg/dL in FH subjects with or without ASCVD enrolled from September 2019 to September 2022 (19, 20). At the last follow-up, LDL-C target was defined as an LDL-C < 55 mg/dL or < 70 mg/dL for FH subjects with or without ASCVD, respectively.

Body weight and height were measured, and body mass index (BMI) was calculated as weight divided by the squared value of height (kg/m2). Arterial hypertension was defined as brachial blood pressure (BP) ≥ 140 mm Hg (systolic) and/or 90 mm Hg (diastolic) on at least two different occasions, or if the subjects were on antihypertensive therapy. Lipid lowering therapy was defined as a daily intake of one of the following drugs: statins, ezetimibe, or PCSK9-i. According to drug intensity, statin therapy was classified as low-intensity (fluvastatin 20–40 mg, lovastatin 20 mg, pravastatin 20 mg, simvastatin 10 mg) moderate-intensity (fluvastatin XL 80 mg, lovastatin 40 mg, pravastatin 40 mg, simvastatin 20–40 mg, atorvastatin 10–20 mg, rosuvastatin 5–10 mg) or high-intensity (atorvastatin 40–80 mg, rosuvastatin 20–40 mg) (21). When they occurred, the fear of statin associated side effects (SASE) or myalgia were reported by FH subjects on low to moderate intensity statins at the last follow-up. PCSK9-mAb therapy included alirocumab or evolocumab. Type 2 diabetes (T2D) was defined as a fasting plasma glucose (FPG) ≥ 126 mg/dL on two consecutive readings and/or glycated hemoglobin (HbA1c) ≥ 6.5% or the use of anti-diabetic medications (22). Smoking habits were divided into either current smoking (defined as a minimum of one cigarette in the last month) or not (23). ASCVD was defined as a documented myocardial infarction, acute coronary syndrome, coronary revascularization (percutaneous coronary intervention or coronary artery bypass graft surgery) or other arterial revascularization procedures, stroke or transient ischemic attack, or peripheral arterial disease (24).

The study population was stratified into two groups according to sex. The study was approved by the local ethics committee in accordance with the ethical standards of the institutional and national research committees and with the 1964 Declaration of Helsinki and its later amendments or comparable ethical standards. Informed consent was obtained from each subject enrolled in the study.

### Biochemical analysis

FPG was measured with the glucose oxidase method. Serum total cholesterol (TC), triglycerides (TG), high-density lipoprotein cholesterol (HDL-C), hs-CRP, aspartate transaminase (AST), alanine transaminase (ALT), and creatine phosophokinase (CPK) were assessed by available enzymatic methods. Apolipoprotein B (ApoB), and Apolipoprotein A1 (ApoA1) were evaluated with a nephelometer assay (Siemens AG Healthcare Sector, Erlangen, Germany). Levels of lipoprotein(a) [Lp(a)] were measured with the latex agglutination immunoassay. LDL-C was calculated using the Friedewald formula. HbA1c was measured with high-performance liquid chromatography using a National Glycohemoglobin Standardization Program and standardized to the Diabetes Control and Complications Trial assay reference (22). Chromatography was performed using a certified automated analyzer (HPLC; HLC-723G7 hemoglobin HPLC analyzer; Tosoh Corp.; normal range 4.25-5.9% [23-41 mmol/mol]).

### Statistical analysis

The distributional characteristics of each variable, including normality, were assessed by the Kolmogorov-Smirnov test. Data are reported as mean ± standard deviation (SD) for continuous parametric and median (interquartile range-IQR) for continuous non-parametric variables and as frequency (percentage) for categorical variables. When necessary, continuous non-parametric variables (TG, Lp(a), hs-CRP, CPK) were logarithmically transformed for statistical analysis to reduce skewness. The Chi square (χ^2^) test was used for categorical variables. To test differences in clinical and biochemical characteristics between the groups Student’s t test was used.

In a secondary analysis, the study population was stratified into four groups according to sex and SLCO1B1 rs4149056 presence: men without SLCO1B1 rs4149056 (M/SLCO1B1- group), women without SLCO1B1 rs4149056 (W/SLCO1B1- group), men with SLCO1B1 rs4149056 (M/SCLO1B1+ group), women with SLCO1B1 rs4149056 (W/SCLO1B1+ group). A χ^2^ test was performed to assess the distributions of high-intensity statins, LDL-C target, fear of SASE and reported myalgia in the four groups. In order to evaluate the impact of sex and SLCO1B1 rs4149056 on LDL-C target achievement, we performed a logistic regression analysis adjusted for age, statin intensity, ezetimibe, and PCSK9-i. The variance inflation factor (VIF) was used to check for the problem of multicollinearity in multivariate analysis. All statistical analyses were performed using IBM SPSS Statistics for Windows version 23. For all tests, *p* < 0.05 was considered significant.

## Results

**Figure 1:**
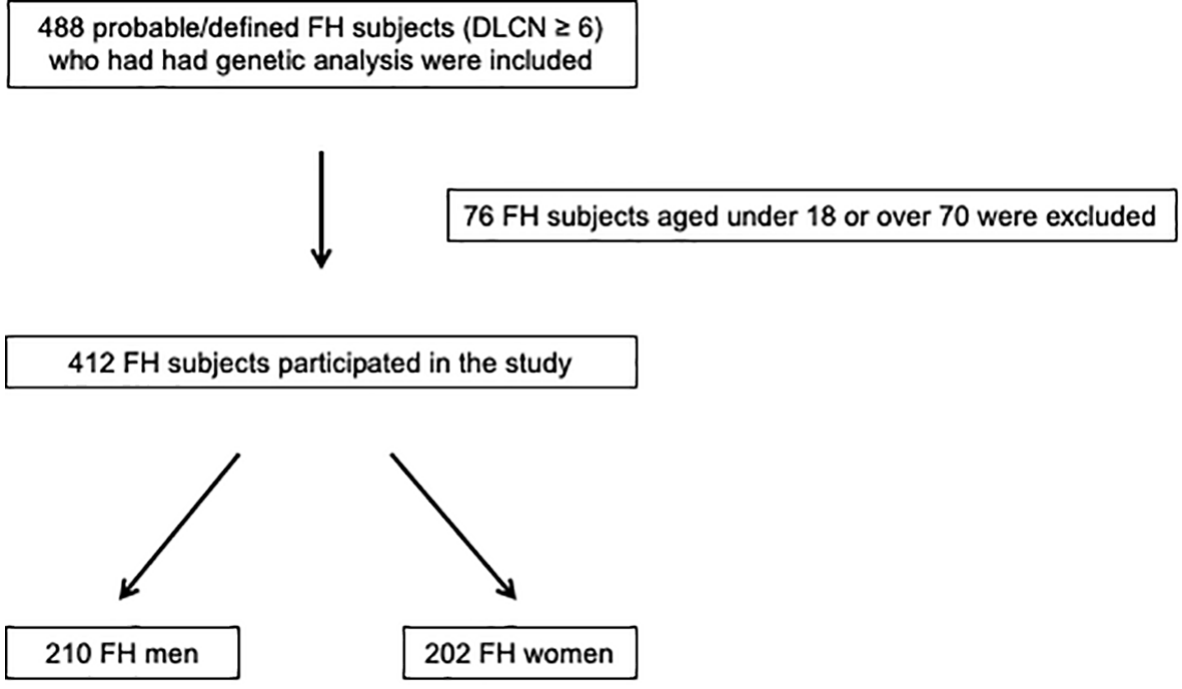
Enrollment flowchart of the study population. DLCN, Dutch Lipid Clinic Network**;** FH, familial hypercholesterolemia.

A total of 488 probable/defined FH subjects who had had genetic analysis were evaluated; of these, 412 FH subjects (210 men and 202 women) satisfied the inclusion criteria and participated in this retrospective observational study (**Figure 1**).

The genetic profile of the study population is presented in **Table 1**. While fewer than one third of subjects did not present a genetic variant, the prevalence of mutation positive FH was 72.6% and it was similar both in men and women. The majority of subjects were heterozygous FH and the most frequent genetic variant was LDLR mutation with no difference between the two groups. Finally, the proportion of FH subjects with SLCO1B1 rs4149056 was 24.5% and it was similar between FH men and women.

**Table 1:** Genetic profile of the Study Population stratified according to sex.

	Men (n = 210)	Women (n = 202)	*p* Value between two groups
FH Genotype			
Mutation-negative, n (%)	58 (27.6)	55 (27.2)	0.87
Mutation-positive, n (%)	152 (72.4)	147 (72.8)	0.87
- LDLR, n (%)	149 (98.0)	144 (97.9)	0.91
*- LDLR defective, n (%)*	83 (55.7)	77 (53.5)	0.23
*- LDLR null, n (%)*	66 (44.3)	67 (46.5)	0.23
- ApoB, n (%)	3 (2.0)	1 (0.7)	–
- PCSK9, n (%)	–	1 (0.7)	–
- ApoE, n (%)	–	1 (0.7)	–
FH Phenotype			
Heterozygous FH, n (%)	147 (98.7)	142 (98.6)	–
Compound heterozygous FH, n (%)	2 (1.3)	1 (0.7)	–
Homozygous FH, n (%)	–	1 (0.7)	–
SLCO1B1 Polymorphism			
rs4149056, n (%)	48 (22.9)	53 (26.2)	0.26
*- Heterozygous rs4149056, n (%)*	47 (97.9)	51 (96.2)	0.72
*- Homozygous rs4149056, n (%)*	1 (2.1)	2 (3.8)	–

Data are presented as mean ± standard deviation or percentages. FH, familial hypercholesterolemia; LDLR, low-density lipoprotein receptor; ApoB, apolipoprotein B; PCSK9, proprotein convertase subtilisin/kexin type 9; ApoE, apolipoprotein E; SLCO1B1, solute carrier organic anion transporter family member 1B1.

**Table 2** shows the baseline characteristics of the study population. No differences in age and BMI were found between the two groups and the prevalence of FH subjects with ASCVD history was similar both in men and women. Pretreatment TC and LDL-C as well as baseline TC, LDL-C, Non-HDL-C, ApoB and Apo AI plasma levels were significantly higher in women compared to men (for pretreatment TC 339.26 ± 26.19 *vs* 356.15 ± 25.97 *p* < 0.05; for pretreatment LDL-C 255.21 ± 24.47 *vs* 271.14 ± 23.87 *p* < 0.05; for TC 217.93 ± 25.71 *vs* 235.52 ± 24.93 *p* < 0.05; for LDL-C 149.54 ± 21.84 *vs* 164.21 ± 21.13 *p* < 0.05; for Non-HDL-C 165.76± 22.08 *vs* 180.69 ± 21.93 *p* < 0.05; for ApoB 114.64 ± 12.44 *vs* 128.22 ± 12.01 *p* < 0.05; for Apo AI 140.65 ± 13.8 *vs* 151.22 ± 12.81 *p* < 0.001). Moreover, the percentage of FH subjects on lipid lowering therapy was significantly lower in women than men (42.6% *vs* 61%, *p* < 0.01); of these, while a higher prevalence of FH subjects on moderate or high-intensity statins were found in men than women (45.0% *vs* 31.9% and 25.4% *vs* 19.1% respectively, for both *p* < 0.05) the distribution of subjects on low-intensity statin was higher in FH women than men (49.0% *vs* 29.6%, *p* < 0.01). As concerns lipid lowering combination therapy, while a higher prevalence of FH men was on moderate intensity statins plus ezetimibe compared to women (39.5% *vs* 32.0%, *p* < 0.05), the percentage of subjects on low intensity statins plus ezetimibe was higher in FH women than men (40.0% *vs* 31.6%, *p* < 0.05).

**Table 2:** Baseline characteristics of the Study Population stratified according to sex.

	Men (n = 210)	Women (n = 202)	*p* Value between two groups
Demographic Characteristics			
Age, years	52.4 ± 8.3	51.9 ± 8.7	0.61
Body mass index, kg/m^2^	25.2 ± 3.2	25.1 ± 3.2	0.83
History of ASCVD, n (%)	39 (18.6)	26 (12.9)	0.07
Glucose Profile			
Type 2 diabetes, n (%)	4 (1.9)	3 (1.5)	–
FPG, mg/dL	89.5 ± 5.4	88.8± 5.4	0.59
HbA1c, %	5.5 ± 0.3	5.5 ± 0.3	0.54
Lipid Profile			
Pretreatment TC, mg/dL	339.3 ± 26.2	356.2 ± 26	< 0.05
Pretreatment LDL-C, mg/dL	255.2 ± 24.5	271.1 ± 23.9	< 0.05
TC, mg/dL	217.9 ± 25.7	235.5 ± 24.9	< 0.05
HDL-C, mg/dL	49.5 ± 9.4	56.4 ± 9.3	< 0.001
Triglycerides, mg/dL	95 (77-120)	87 (73-115)	0.06
LDL-C, mg/dL	149.5 ± 21.8	164.2 ± 21.1	< 0.05
Non-HDL-C, mg/dL	165.8 ± 22.1	180.7 ± 21.9	< 0.05
ApoB, mg/dL	114.6 ± 12.4	128.2 ± 12	< 0.05
ApoAI, m g/dL	140.7 ± 13.8	151.2 ± 12.8	< 0.001
ApoB to ApoAI ratio	0.8 ± 0.2	0.9 ± 0.3	0.14
Lp(a), mg/dL	19.9 (10.4-40.4)	22.7 (10.4-45.1)	0.19
LDL-C target, n (%)	11 (5.2)	7 (3.5)	0.11
Liver and Muscle Enzymes			
AST, U/L	25.1 ± 6.9	24.4 ± 7	0.18
ALT, U/L	27.7 ± 8.4	25.8 ± 8.6	0.21
CPK, U/L	126 (94-166)	121 (92-161.5)	0.17
Risk Factors			
Systolic BP, mmHg	120 ± 9.9	118.6 ± 9.8	0.39
Diastolic BP, mmHg	72.1 ± 9.1	70.6 ± 9.5	0.28
Smoking, n (%)	53 (25.2)	42 (20.8)	0.11
hs-CRP, mg/dL	0.1 (0.1-0.2)	0.1 (0.1-0.2)	0.51
Treatment			
Antihypertensive therapy, n (%)	56 (26.6)	45 (22.3)	0.12
Lipid lowering therapy, n (%)	128 (61.0)	86 (42.6)	< 0.01
Statin monotherapy, n (%)	71 (33.8)	47 (23.3)	< 0.01
- *Low-intensity statin, n (%)*	21 (29.6)	23 (49.0)	< 0.01
- *Moderate-intensity statin, n (%)*	32 (45.0)	15 (31.9)	< 0.05
- *High-intensity statin, n (%)*	18 (25.4)	9 (19.1)	< 0.05
Ezetimibe monotherapy, n (%)	19 (9.0)	14 (6.9)	0.13
Statin plus ezetimibe, n (%)	38 (18.1)	25 (12.4)	< 0.05
- *Low-intensity statin plus ezetimibe, n (%)*	12 (31.6)	10 (40.0)	< 0.05
- *Moderate-intensity statin plus ezetimibe, n (%)*	15 (39.5)	8 (32.0)	< 0.05
- *High-intensity statin plus ezetimibe, n (%)*	11 (28.9)	7 (28.0)	0.51

Data are presented as mean ± standard deviation, percentages, or median (interquartile range). ASCVD, atherosclerotic cardiovascular disease; FPG, fasting plasma glucose; HbA1c, glycated hemoglobin; TC, total cholesterol; HDL-C, high-density lipoprotein cholesterol; LDL-C, low-density lipoprotein cholesterol; ApoB, apolipoprotein B; ApoAI, apolipoprotein AI; Lp(a), lipoprotein (a); BP, blood pressure; hs-CRP, high sensitivity C-reactive protein.

At the last follow-up, after LLT optimization, a significant improvement of lipid profile was observed in the study population; however, FH women exhibited a higher LDL-C than men (104.82 ± 20.05 *vs* 92.83 ± 19.79, *p* < 0.05) and the proportion of subjects on LDL-C target was lower in FH women than men (27.7% *vs* 38.1%, *p* < 0.05). The glucose profile was similar between FH men and women and only 3 new cases of T2D occurred. All FH subjects were on lipid lowering therapy but the majority of them were on low to moderate intensity statins and they were more prevalent in FH women than men (70.3% *vs* 48.6% *p* < 0.001). Among these, the percentages of FH subjects who reported the fear of SASE or myalgia were higher in women than men (48.0% *vs* 32.9%, p < 0.01 and 22.3% *vs* 15.7%, p < 0.05, respectively). While a higher prevalence of FH men were on high-intensity statins plus ezetimibe compared to women (53.7% *vs* 32.0%, *p* < 0.01), an increased percentage of subjects on low-intensity statins plus ezetimibe was found in FH women than men (30.5% *vs* 13.2%, *p* < 0.01) and the same prevalence was reported in subjects on statins plus ezetimibe plus PCSK9i (for high-intensity statins plus ezetimibe plus PCSK9i 51.5% *vs* 29.2%, *p* < 0.01; for low-intensity statins plus ezetimibe plus PCSK9i 33.9% *vs* 10.3%, *p* < 0.01) (**Table 3**).

**Table 3:** Metabolic profile and adverse events of the Study Population stratified according to sex at the last follow-up after lipid lowering therapy optimization.

	Men (n = 210)	Women (n = 202)	*p* Value between two groups
Glucose Profile			
FPG, mg/dL	92.5 ± 5.4	90.4 ± 5.8	0.39
HbA1c, %	5.6 ± 0.3	5.6 ± 0.3	0.24
Lipid Profile			
TC, mg/dL	164.6 ± 20.9	175.2 ± 21.3	< 0.05
HDL-C, mg/dL	50.5 ± 9.8	57.2 ± 9.7	< 0.001
Triglycerides, mg/dL	89 (73-115)	82 (66-110)	0.07
LDL-C, mg/dL	92.8 ± 19.8	104.8 ± 20.1	< 0.05
Non-HDL-C, mg/dL	122.5 ± 18.6	124.1 ± 19.5	0.46
ApoB, mg/dL	83.7 ± 13.2	87 ± 12.6	0.38
ApoAI, m g/dL	141.7 ± 13.8	154.9 ± 13.6	< 0.001
ApoB to ApoAI ratio	0.7 ± 0.3	0.7 ± 0.3	0.44
Lp(a), mg/dL	22.4 (10.4-42.5)	26.9 (10.4-51.4)	0.1
LDL-C target, n (%)	80 (38.1)	56 (27.7)	< 0.05
Liver and Muscle Enzymes			
AST, U/L	25.2 ± 6.7	25.5 ± 7.2	0.67
ALT, U/L	27.9 ± 9.5	28 ± 9.9	0.74
CPK, U/L	130 (99-171.5)	144 (107.5-186)	0.09
Treatment			
Lipid lowering therapy, n (%)	210 (100.0)	202 (100.0)	–
High-intensity statin, n (%)	108 (51.4)	60 (29.7)	< 0.001
Low-to-moderate-intensity statin, n (%)	102 (48.6)	142 (70.3)	< 0.001
*- Fear of SASE, n (%)*	69 (32.9)	97 (48.0)	< 0.01
*- Myalgia, n (%)*	33 (15.7)	45 (22.3)	< 0.05
Statin plus ezetimibe, n (%)	136 (64.8)	128 (63.4)	0.82
- *Low-intensity statin plus ezetimibe, n (%)*	18 (13.2)	39 (30.5)	< 0.01
- *Moderate-intensity statin plus ezetimibe, n (%)*	45 (33.1)	48 (37.5)	0.09
- *High-intensity statin plus ezetimibe, n (%)*	73 (53.7)	41 (32.0)	< 0.01
Statin plus ezetimibe plus PCSK9i, n (%)	68 (32.4)	65 (32.2)	0.92
- *Low-intensity statin plus ezetimibe plus PCSK9i, n (%)*	7 (10.3)	22 (33.9)	< 0.01
- *Moderate-intensity statin plus ezetimibe plus PCSK9i, n (%)*	26 (38.2)	24 (36.9)	0.37
- *High-intensity statin plus ezetimibe plus PCSK9i, n (%)*	35 (51.5)	19 (29.2)	< 0.01
Ezetimibe plus PCSK9i, n (%)	6 (2.8)	9 (4.4)	0.12
Adverse events			
Newly diagnosed T2D, n (%)	2 (1.0)	1 (0.5)	–
Newly diagnosed cardiovascular events, n (%)	5 (2.4)	7 (3.5)	0.36

Data are presented as mean ± standard deviation, percentages, or median (interquartile range). ASCVD, atherosclerotic cardiovascular disease; FPG, fasting plasma glucose; HbA1c, glycated hemoglobin; TC, total cholesterol; HDL-C, high-density lipoprotein cholesterol; LDL-C, low-density lipoprotein cholesterol; ApoB, apolipoprotein B; ApoAI, apolipoprotein AI; Lp(a), lipoprotein (a); BP, blood pressure; hs-CRP, high sensitivity C-reactive protein; SASE, statin associated side effects; PCSK9-i, proprotein convertase subtilisin/kexin type 9 inhibitors; T2D, type 2 diabetes.

**Figure 2:**
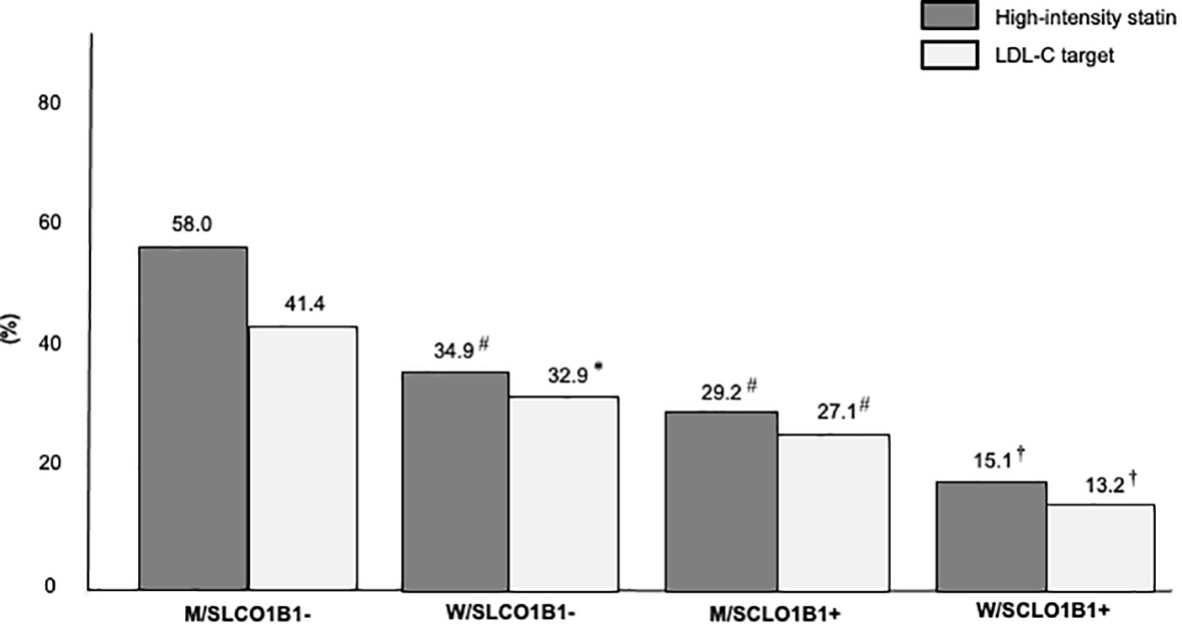
Percentages of high-intensity statin use and LDL-C target achievement in the Study Population stratified according to sex and SLCO1B1 rs4149056 presence after lipid lowering therapy optimization. M/SLCO1B1-, men without SLCO1B1 rs4149056; W/SLCO1B1-, women without SLCO1B1 rs4149056; M/SCLO1B1+, men with SLCO1B1 rs4149056; W/SCLO1B1+, women with SLCO1B1 rs4149056. *, *p <*0.05; #, *p* < 0.01; †, *p* < 0.001.

**Figure 3:**
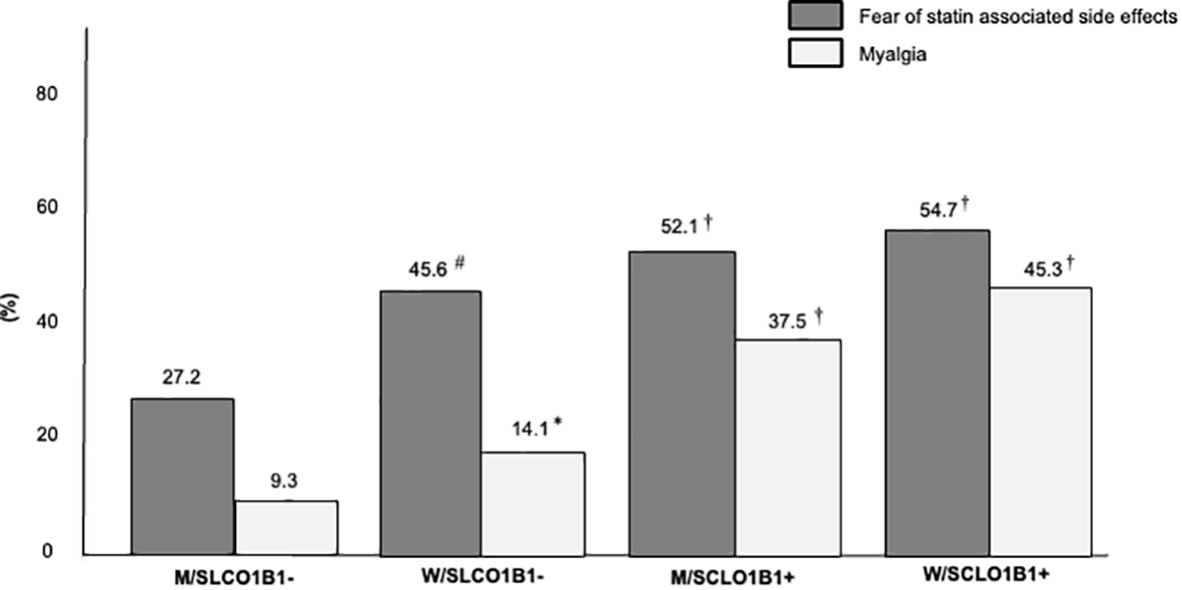
Percentages of FH subjects with fear of statin associated side effects and reported myalgia in the Study Population stratified according to sex and SLCO1B1 rs4149056 presence after lipid lowering therapy optimization. M/SLCO1B1-, men without SLCO1B1 rs4149056; W/SLCO1B1-, women without SLCO1B1 rs4149056; M/SCLO1B1+, men with SLCO1B1 rs4149056; W/SCLO1B1+, women with SLCO1B1 rs4149056. *, *p <*0.05; #, *p* < 0.01; †, *p* < 0.001.

In a secondary analysis, the study population was stratified into four groups according to sex and SLCO1B1 rs4149056 presence: men without SLCO1B1 rs4149056 (M/SLCO1B1- group), women without SLCO1B1 rs4149056 (W/SLCO1B1- group), men with SLCO1B1 rs4149056 (M/SCLO1B1+ group), women with SLCO1B1 rs4149056 (W/SCLO1B1+ group). After LLT optimization, the percentage of FH subjects on high-intensity statins decreased from the M/SLCO1B1- group to the W/SLCO1B1+ group and the same was found in LDL-C target distribution (for both *p* for trend < 0.01) (**Figure 2**); however, the prevalence of SASE fear increased from the M/SLCO1B1- group to the W/SLCO1B1+ group and the same was observed in reported myalgia distribution (for both *p* for trend < 0.01) (**Figure 3**).

Logistic regression analysis showed that the W/SCLO1B1-, M/SCLO1B1+ and W/SCLO1B1+ groups were inversely associated with LDL-C target achievement (*p* for trend < 0.001) and the F/SCLO1B1+ group exhibited the strongest association (**Table 4**).

**Table 4:** Logistic regression of LDL-C Target Achievement in the Study Population stratified according to sex and SLCO1B1 rs4149056 presence.

Quartiles	No. of Participants	Multivariate ORs (95% CIs)
		Model
M/SLCO1B1-	162	1.00 (reference)
W/SLCO1B1-	149	0.72 (0.6 – 0.85)
M/SLCO1B1+	48	0.69 (0.58 – 0.81)
W/SLCO1B1+	53	0.39 (0.3 – 0.52)
*P* for trend		< 0.001

Logistic regression model was used to estimate ORs and 95% CIs. The model was adjusted for age, statin intensity, ezetimibe, and PCSK9-i. M/SLCO1B1-, men without SLCO1B1 rs4149056; W/SLCO1B1-, women without SLCO1B1 rs4149056; M/SCLO1B1+, men with SLCO1B1 rs4149056; W/SCLO1B1+, women with SLCO1B1 rs4149056.

## Discussion

In this study, we investigated the impact of SLCO1B1 rs4149056 on LDL-C target achievement after lipid lowering therapy optimization in FH men and women; to the best of our knowledge, this is the first study exploring the SLCO1B1 genotype-sex interaction in this population. We found that the prevalence of subjects on LDL-C target as well as on high intensity statin therapy was significantly lower in FH women with SLCO1B1 rs4149056 than the other groups; moreover, FH women with SLCO1B1 rs4149056 exhibited the strongest inverse association with LDL-C target. Thus, our findings suggest that the genotype effect of SLCO1B1 rs4149056 could be more pronounced in women than men and it is in line with a previous finding by Turkmen et al. who found that, in a large cohort of subjects in a primary care setting, women with SLCO1B1 rs4149056 had elevated cholesterol levels compared to men and this was largely explained by a higher prevalence of women who discontinued the prescribed statins (25). A possible explanation of this finding may be that the sex difference of SLCO1B1 rs4149056 effect could be due to biological differences as well as to a lower percentage of muscle mass in women than men leading to an increased plasma level of statins with a possible higher risk of muscle symptoms (26). In this context, in our study a higher prevalence of myalgia as well as of SASE fear were observed in FH women with SLCO1B1 rs4149056 than men with the same polymorphism or subjects without SLCO1B1 rs4149056. Our results are in line with previous findings that evaluated the sex difference of statin therapy management in the general population (27, 28). In fact, Voora et al. found that in the STRENGHT Study SASE were more prevalent in women than men and that SLCO1B1 rs4149056 and female sex were significantly associated with SASE; moreover, Bradley et al. showed that in the PALM Registry the fear of side effects was the main reason for statin discontinuation and this was largely observed in women. However, in our study we found that after lipid lowering therapy optimization the majority of FH women were on low to moderate intensity statins plus other lipid lowering drugs and this was in line with a recent finding by Schreuder et al. who found that in a multicenter cohort of FH subjects, more than half of the women were on low to moderate intensity statins and only 26.9% of them achieved the recommended LDL-C target (29). Accordingly, in our study after lipid lowering therapy optimization the percentage of FH women who reached the specified LDL-C target was 27.7%.

In the last few years, it has been shown that in FH the cardiovascular risk is heterogeneous and the identification of FH subjects who are more vulnerable to cardiovascular injury is needed to better improve their management and treatment (30, 31). In this context, sex related differences of cardiovascular prevention and LLT management have been observed in FH subjects (32, 33); these findings could have a deleterious impact on the long-term cardiovascular health in this population. This could be attributable to different behavioral characteristics, life course lipoprotein distribution or hormone related lipid fluctuations (34, 35); however, genetic polymorphisms involved in pharmacokinetic and pharmacodynamic pathways could also influence the sex differences of LLT adherence (14, 36). In this context, in our study the genetic evaluation of SLCO1B1 rs4149056 presence was able to detect FH subjects who reported myalgia, discontinued high intensity statins and did not achieve the recommended LDL-C target. Thus, the application of a genetic tool able to identify subjects at higher risk of statin intolerance could be useful to ameliorate LLT management in FH subjects more vulnerable to cardiovascular injury (37).

There are several limitations to our study; first this was a retrospective observational study and thus causal relationship and temporality cannot be established between starting lipid lowering therapy optimization and reported myalgia or SASE fear. Moreover, based on the type of study the lipid lowering therapy optimization after the addition of inclisiran or bempedoic acid was not evaluated due to the restricted time of follow-up. Furthermore, no data on muscle mass as well as on plasma levels of sex hormones, menopausal status or estrogen supplementation were available in our cohort of subjects; further studies are needed to better evaluate the impact of these variables on lipid lowering therapy optimization in FH subjects with SLCO1B1 rs4149056. Finally, data on nutritional counseling as well as on physical activity were not available.

In conclusion, the adherence of intensive lipid lowering therapy was low in FH women with SLCO1B1 rs4149056 and these subjects rarely achieved the recommended LDL-C target in clinical practice. The genotype effect of SLCO1B1 rs4149056 could be more pronounced in women than men; further prospective studies are needed to evaluate the applicability of a genetic tool able to identify FH subjects who are more vulnerable to cardiovascular injury.

## Data availability statement

The original contributions presented in the study are included in the article/supplementary material, further inquiries can be directed to the corresponding author/s.

## Ethics statement

The studies involving humans were approved by Catania 2, Piazza Santa Maria di Gesù n° 5, Catania, Italy. The studies were conducted in accordance with the local legislation and institutional requirements. The participants provided their written informed consent to participate in this study.

## Author contributions

GB: Conceptualization, Data curation, Methodology, Writing – original draft, Software. FB: Data curation, Investigation, Writing – review & editing. MD: Data curation, Investigation, Writing – review & editing. NM: Data curation, Investigation, Writing – review & editing. SSc: Data curation, Investigation, Writing – review & editing. SSp: Data curation, Investigation, Writing – review & editing. AV: Data curation, Investigation, Writing – review & editing. FD: Data curation, Investigation, Writing – review & editing. MM: Data curation, Investigation, Writing – review & editing. SD: Data curation, Investigation, Writing – review & editing. AF: Data curation, Investigation, Writing – review & editing. AS: Data curation, Investigation, Writing – review & editing. AM: Data curation, Investigation, Methodology, Visualization, Writing – review & editing. AD: Data curation, Investigation, Methodology, Visualization, Writing – review & editing. LF: Data curation, Investigation, Methodology, Visualization, Writing – review & editing. FP: Data curation, Investigation, Methodology, Visualization, Writing – review & editing. SP: Conceptualization, Data curation, Funding acquisition, Investigation, Methodology, Supervision, Validation, Visualization, Writing – review & editing. RS: Conceptualization, Data curation, Investigation, Methodology, Software, Supervision, Validation, Visualization, Writing – review & editing.
